# Holding together industrial time: Coordinated temporalities on a dairy farm in China

**DOI:** 10.1177/0961463X251395784

**Published:** 2025-11-20

**Authors:** Wenjia Zhou

**Affiliations:** 1 Department of Interdisciplinary Studies of Culture, 8018 Norwegian University of Science and Technology, Trondheim, Norway

**Keywords:** China, industrial agriculture, more-than-human, temporality, temporal coordination

## Abstract

This paper examines the temporal organization of industrial dairy production in China. Based on ethnographic fieldwork on a small-scale farm in Hebei Province, it showcases how a seemingly seamless 24/7 milk production cycle is sustained through the coordination of multiple, divergent temporalities. These include reproductive rhythms shaped by both bovine biology and market dynamics, the processual temporality of care and repair, and the agricultural temporality rooted in the 24 solar terms that structure traditional farming cycles in China. Engaging with theoretical work on temporal multiplicity and coordination, this paper highlights the costs of sustaining industrial production time, borne disproportionately by cows, human workers, ecologies, and small farms. By making visible the embodied and more-than-human labor required to sustain industrial time, this paper contributes to current discussions to rethink modernity through time. It offers an empirically grounded account of how divergent rhythms are held together through friction, improvisation, and care in industrial agriculture, calling for a temporal ethic grounded in responsiveness, care, and relational coordination.

## Introduction

In July 2024, at the 15th China Dairy Conference, industry leaders described China's dairy sector as facing its most severe crisis since 2008, with years of steady production growth giving way to a growing imbalance between supply and demand. In response, solutions proposed at the conference centered on further expanding demand and enhancing efficiency through technological innovation rather than questioning overproduction. This persistent emphasis on growth is deeply rooted in the historical development of China's dairy sector, where milk production and consumption have long been tied to broader national goals of health and strength since the Republican era (Lu, 2020). Over time, demand for milk has grown through state promotion, industrial expansion, and market campaigns by major dairy firms. Policies have favored consolidation within a handful of dominant companies and promoted capital-intensive processing. Meanwhile, consumer preference for standardized, branded products has further reinforced this shift. These forces have normalized dairy consumption as a marker of safety and middle-class modernity, reinforcing a development path of accelerated scale and production (DuBois, 2022; Mak, 2021; Smith, 2019). In recent decades, domestic dairy consumption has surged, tripling between 2000 and 2010 (Smith, 2019), which has made China one of the world's largest dairy importers. Alongside this, domestic milk production has steadily increased, reaching approximately 30.5 million tons in 2023, with a compound annual growth rate of 1.5% from 2012 to 2023 (Huld, 2024). To sustain this momentum, the Chinese government has introduced a series of national policies aimed at revitalizing the dairy sector. The 2022 “Five-Year Plan to Enhance Dairy Industry Competitiveness” projected total output to reach 41 million tons by 2025, with 75% of dairy farms housing over 100 cows, thus meeting the threshold for intensive farming.

Yet amid this rapid expansion, the industry is facing a slowdown in consumption, creating a widening gap between supply and demand. In 2023, China's milk output reached 42.81 million tons, up 6.3% year-on-year, while overall dairy consumption declined by approximately 1.6%, reflecting the combined effects of the pandemic, slowing economic growth, and declining household incomes (Shen, 2024). This mismatch signals a broader crisis in the industry, which experts link to the cyclical nature of agricultural commodities such as pork (Coase and Fowler, 1937; Dawson, 2009). As Xia et al. (2023) note, China's dairy sector typically undergoes an 8- to 9-year cycle, alternating between 4 and 5 years of growth and 3 and 4 years of adjustment. As agricultural economist Liu (2025) observes, the current downturn stems from structural contradictions on the supply side: while large-scale farms continue to expand, small- and medium-sized social farms are being steadily forced out, undermining the traditional market mechanisms that once balanced supply and demand. In recent years, capitalized dairy enterprises have consolidated the sector by investing in and controlling large-scale farms housing thousands to over ten thousand cows. Against this backdrop, herds of 200–500 cows, typically run as social farms without corporate backing, are now regarded as small- and medium-scale, despite once being considered substantial. In practice, farms smaller than this struggle to survive within the industrial supply chain at all. These social farms face limited access to capital and labor, and their disadvantage is further compounded by differential milk pricing: dairy companies prioritize milk from their own or affiliated large-scale farms at higher purchase prices, while milk from social farms is discounted. Lacking stabilizing mechanisms such as the European Union's quota system and milk package to address the structural milk oversupply (European Commission, 2016), these farms are left vulnerable to price collapses and cost surges. Meanwhile, state subsidies and policy support are increasingly concentrated on capital-intensive large farms, leaving smaller farms further marginalized. As Liu (2025) argues, the survival of these small- and medium-sized social farms is essential not only for equity, but also for the long-term resilience of the industry, underlining these farms’ roles in environmental sustainability and rural livelihoods.

This paper focuses on such a small-scale farm owned by Ailin in rural Shijiazhuang, Hebei Province, that I encountered during my doctoral fieldwork for studying China's dairy industry. Between December 2023 and August 2024, I visited dairy operations of various scales: from households in Yunnan raising fewer than 20 cows, to small- and medium-sized social farms with 700 cows in North China, and large corporate-owned operations with herds of over 10,000. I interviewed actors across the dairy industry and attended agriculture- and dairy-related conferences. In March 2024, I met Ailin at an agricultural conference in Beijing and subsequently stayed at her farm for 1 week. I returned for two visits in May and June, each lasting 2 weeks. During these stays, I lived with Ailin's family, socialized with her friends and a local dairy company's representatives, and shadowed farm workers’ daily routines while occasionally assisting with tasks, after obtaining informed consent from all participants. To prevent potential implications for participants’ livelihoods and their professional ties with the dairy company, all personal names, the dairy company, and sub-municipal locations are anonymized.

Located in rural Hebei, one of North China's major dairy-producing hubs, Ailin's farm operates within an intensive industrial landscape. In 2023, Hebei ranked second nationwide with a dairy herd of 1.51 million and a reported 100% intensive farming rate (Hao, 2024). By 2019, 98.2% of the herd was concentrated on farms with more than 300 cows. With 360 cows in 2022, Ailin's farm ranked near the lower end of the county's 70 dairy farms: while the smallest had around 200 cows, 11 exceeded 1000, including one with 4500. Ailin's farm stands out as both small in scale by local standards and industrial in practice. It supplies milk to a major dairy company, employs mechanized milking and formulated feed, yet remains marginal due to its limited size, exclusion from subsidies, and heightened exposure to market risks. Following Tsing's (1994: 279) framing of margins as “zones of unpredictability” where discrepant forms of meaning-making converge, I approach the farm's marginal position not as a disadvantage but as an analytical vantage point that exposes how different logics of coordination converge at the edges of industrial systems. During my fieldwork, I observed milk production unfold through daily routines—feeding, milking, cleaning—performed with clockwork regularity. Yet, I also encountered moments when these rhythms faltered, making visible the frictions and ongoing adjustments required to sustain production. The contrast between these temporal registers drew my attention to temporality itself, raising questions about how industrial temporality is not simply imposed from above, but constructed through the ongoing coordination of animals, humans, machines, and seasons. Compared to larger and more mechanized farms, Ailin's farm makes these tensions particularly visible. Its peripheral position within the dairy system enabled both a unique perspective on these diverse rhythms and a level of ethnographic access rarely possible in larger, more restricted farms.

Drawing on the daily operations of Ailin's farm, this paper examines how industrial dairy production in China is temporally organized to sustain its growth-oriented trajectory. While the farm's vulnerability reflects the precarity of small- and medium-scale operations, it fundamentally points to a structural temporal fragility at the heart of industrial agriculture itself—one that arises from the relentless demands of acceleration. China's dairy industry is shaped by a vision of progress rooted in growth, expansion, and technological efficiency, and deeply intertwined with the state's broader goals of national modernization. This resonates with broader critiques of modern industrial societies, where speed is not merely a result of development but an organizing principle in itself (Harvey, 1990; Hassan, 2009; Rosa, 2003, 2013; Sewell, 2008; Virilio, 1986; Wajcman, 2014). By defining acceleration as “quantitative growth per unit of time,” Rosa (2013: 160) underlines that acceleration is fundamentally about time. The time structures of modernity are built upon the imperative of acceleration, where production, distribution, and consumption must continuously intensify to sustain the logic of economic growth. Within this logic, acceleration is framed as a solution to its own crises in industrial agriculture. Adam (1998) critiques this reductionist framing of time in industrial agriculture, arguing that it compresses complex ecological and social rhythms into linear, abstract metrics of efficiency. Her timescape perspective invites us to understand time as relational and multilayered, where social–environmental processes unfold through overlapping and often conflicting rhythms. Attending to these temporalities, as Rose (2013) and Tsing (2015) remind us, is not only analytical but also ethical and political, particularly in the face of ecological precarity and multispecies interdependence.

Rather than understanding time as a fixed structure, I treat it as something that emerges from situated practices and interwoven rhythms among more-than-human actors (Gan, 2017a; Griffiths, 2023; Ingold, 2000; Sharma, 2014). The concept of more-than-human underlines how human lives are “always already” entangled with the material worlds of which it is a part (Lorimer and Hodgetts, 2024: 13), and the formation of worlds always involves a multiplicity of actors and agencies beyond the human (Price and Chao, 2023). Taking into account material agency and relational processes, I ask: How is industrial dairy production temporally organized on Ailin's farm, and what forms of coordination make its rhythms possible? Rather than assuming a seamless, self-sustaining system, I trace how different temporal rhythms, such as biological, mechanical, and agricultural rhythms, are actively synchronized, negotiated, and sometimes disrupted, with workers and cows continually adjusting to maintain production. This focus highlights the costs at which such coordination is achieved, as disruptions expose the hidden labor and vulnerabilities that underpin the system. In doing so, the paper contributes a rare ethnographic account of how industrial time is assembled on a small-scale farm in China, extending discussions on more-than-human temporal coordination by foregrounding the vulnerable, and at times expendable, life forms that bear the costs of pursuing efficiency.

In what follows, I begin by reviewing the literature on temporality, highlighting how time is relational, embodied, and coordinated across human and more-than-human actors. I then turn to Ailin's dairy farm, situating it as a marginal actor within the industrial dairy landscape before analyzing the multiple temporalities that structure everyday production. I conclude by tracing how temporal coordination sustains industrial production while exposing the contradictions and hidden costs of acceleration at the heart of industrial modernity.

## Coordinated temporalities in a more-than-human world

In this paper, time is not seen as a static or pre-existing entity; rather, it is a “symbolic process continually produced in everyday practices” (Munn, 1992: 116). Time is not merely a neutral backdrop to more-than-human activities; rather, it is intrinsically entangled with material processes, shaping and being shaped by the ways in which forms and practices come into being within diverse ecosystems (Barad, 2007: 180). Temporality, as Gan (2017a: 90) describes, is “a series of coordinations across incommensurabilities or qualitatively different ontologies.” Crucially, these coordinations are neither random nor natural; they emerge historically through intersecting relations shaped by political, economic, moral, and material contexts (Sharma, 2014; Shove et al., 2009). This perception of time as relational, embodied, and historically produced provides a lens to examine how industrial capitalism profoundly shapes temporal experiences and relations.

The rise of industrial modernity introduced a normative conception of time that is abstract, linear, and progressive, closely tied to the acceleration of technological and social processes (Elias, 2007; Nowotny, 1994; Rosa, 2013). As Thompson (1967) famously argues, the rise of industrial society brought about a fundamental restructuring of work habits, imposing new disciplines, incentives, and ways of experiencing time. This transformation is not merely conceptual but deeply embodied, so that workers came to internalize the time of the factory, where efficiency and productivity were prioritized over the rhythms of nature and the body.

Yet, the abstraction of modern time is fundamentally incompatible with the multiplicity of local temporalities in more-than-human worlds. While industrial production is organized around a single, uniform tempo of acceleration and growth, bodies follow their own biological rhythms (Singleton, 2010), while ecosystems such as forests (Auerbach and Clark, 2018), soils (de la Bellacasa, 2015), water bodies (Jensen, 2024; Probyn, 2016), and countless other entities all operate on vastly different timescales. The imposition of a linear, modernist temporality not only disrupts these rhythms but also marginalizes their significance in favor of a singular, hegemonic view of the present and future, that is always oriented toward progress, growth, and expansion (Adam, 1990; Tsing, 2015).

Thompson's (1967) comparison of industrial time with Evans-Pritchard's (1940) account of the Nuer people's “cattle clock” highlights this contrast. Industrial capitalism abstracts time into measurable units, oriented toward productivity, efficiency, and punctuality. While for the Nuer, time emerges from within the flow of daily life. Milking, herding, and byre cleaning simply follow the rhythms of the cattle, the weather, and the seasons. In this system, time is cyclical and situated, shaped by ecological and social relations rather than externally imposed. The cattle clock resonates with Ingold's (1993) concept of the taskscape, where time emerges through the ongoing, mutually attentive performance of tasks. Drawing an analogy between orchestral music and social life, Ingold argues that sociality itself is founded on the resonance of movements and feelings generated from shared practices. Ingold (1993: 160) sees that “music mirrors the temporal form of the taskscape,” where cycles and repetitions are rhythmic instead of metronomic, forming a complex interweaving of multiple concurrent cycles in motion.

Gan and Tsing (2018) extend the taskscape concept to explore the turbulence and incommensurabilities within more-than-human assemblages, understood as an ongoing practice of “becoming with,” a continuous unfolding in which humans and non-humans align through temporal co-constitution. They describe temporal coordination not as a seamless orchestral unity but as a baroque fugue, where separate melodic lines intertwine, producing moments of harmony and dissonance as they cross. This resonates with Lefebvre's (2004: 16) typology of rhythm, in which eurhythmia, arrhythmia, and polyrhythmia describe varying degrees of coordination, misalignment, and coexistence among divergent temporalities. Such a perspective not only captures the contingent nature of coordination within more-than-human assemblages but also reveals a logic that underpins industrial systems. As Tsing (2015: 24) argues, industrial production is not driven by a singular, uninterrupted temporal flow, but relies on the coordination of overlapping rhythms. These rhythms rarely align and require continuous work to navigate frictions. At the margins of industrial systems, such alternative temporalities become especially visible, not as oppositions to industrial time, but as its necessary components.

Recognizing these polyphonic interactions is crucial for understanding how industrial assemblages persist—a recognition that also carries important ethical implications. Industrial acceleration, agricultural intensification, and market-driven production cycles compress time and extract value in ways that disrupt the entangled temporalities of more-than-human worlds (Adam, 1998; Gan, 2017a). As Gan (2017b) argues, lively bodies and landscapes are often traded and taxed to secure imagined futures. In this paper, I aim to uncover the alternative temporalities that are obscured by what Lipscomb (2011: 284) describes as “the seamless flow of our capitalist-bureaucratic experience of time,” asking whose rhythms are sustained, whose are disrupted, and who bears the costs of these reorganizations. Drawing on Ailin's farm, I approach the site not as a space of linear industrial production, but as a situated, more-than-human assemblage held together through ongoing temporal negotiations. Attending to these multiple and often conflicting temporalities—industrial, biological, mechanical, and environmental—reveals how production is sustained through continuous acts of coordination. Meanwhile, it foregrounds the uneven distribution of burdens when dominant temporal logics override others. Before introducing the diverse temporalities at play on the farm, I offer a brief overview of the farm's history and current operational challenges, which reflect broader tensions within China's dairy sector.

## Ailin's dairy farm

Ailin's farm sits on the outskirts of Shijiazhuang, the capital of Hebei Province at 38 °N latitude. Owing to its temperate climate and abundant corn production, Hebei has long been a key hub for China's dairy industry (Fan et al., 2020). Shijiazhuang notably housed the former headquarters of the Sanlu Group, once a major player in the sector. In 2008, Sanlu was found to have sold contaminated milk products that caused widespread health issues, particularly among infants. The scandal, which led to the collapse of Sanlu, not only devastated consumer trust in domestic dairy products but also severely affected household farmers whose livelihoods were tied to the dairy industry across China. In its aftermath, dairy companies stopped purchasing milk directly from individual farmers. In response, cow owners in Ailin's village formed a cooperative for collective feeding, milking, and management. Over the past decade, Ailin's father and Ailin gradually acquired all the cows, transforming the cooperative into a family-run farm. Ever since the establishment of the farm, they have been in cooperation with Dingyao Dairy, the largest dairy company after Sanlu in Hebei. As of early 2024, the farm maintained approximately 600 cows, placing it at the lower end of Dingyao's partner farm scale.

At the beginning of 2024, Ailin renewed a 5-year contract with Dingyao. This contract ensures that Dingyao purchases milk produced on her farm for the next 5 years. In line with national revitalization goals, Hebei's Department of Agriculture and Rural Affairs launched a 2022 action plan with 10 key tasks to boost dairy competitiveness. Among them was the promotion of long-term purchase contracts between dairy companies and farms, which may have helped sustain Ailin's cooperation with Dingyao during the period of raw milk oversupply. However, the contract does not guarantee that all the milk from Ailin's farm will be collected, and the collected milk is typically purchased at a lower price than that from company-owned farms, leaving social farms like hers at a clear disadvantage. Starting in February 2024, Dingyao introduced “分流 *fenliu* (diversion)” as a way of managing milk oversupply, which practically means not buying all the milk from partner social farms. The term “分流 *fenliu*” previously appeared during the wave of layoffs in China's state-owned enterprise (SOE) reforms in the 1990s as a strategy for restructuring labor relations in a more effective way. Unlike “下岗 *xiagang* (laid-off)” workers, diverted workers remained formally employed but were reassigned, often to outsourced roles, with reduced wages and benefits (Solinger, 2001). While the 1990s layoffs and diversions aimed to address SOE overstaffing, the 2024 dairy diversions respond to milk oversupply.

For dairy farmers, being “diverted” means that not all their milk is collected, forcing them to manage the surplus by producing milk powder, selling to different buyers, or even discarding it in extreme cases. In February, more than 4 tons of milk from Ailin's farm was diverted. In May, the amount of diverted milk increased to 36 tons. Dingyao provides factory access for small farms to spray-dry excess milk into powder for a fee but leaves them responsible for finding their own buyers. The measures to divert excess milk are not unique to Dingyao alone. Dairy farms I visited in Hohhot, Inner Mongolia, in March 2024 also faced similar issues. Boss Long, a local farm owner, had to sell milk from his high-yield cows to peddlers at an extremely low price. He told me, “Cows must eat and be milked every day. Not a single day can be skipped.” 

Despite market fluctuations, dairy cows’ biological rhythms on the farm persist. In the next section, I will reveal the overlapping rhythms that structure production: the rigid industrial schedule, the cows’ calibrated biological cycles, the non-linear process time of care and repair, and seasonal rhythms. Their interweaving sustains daily operations, but only through constant adjustments, the costs of which are borne by workers, cows, and even the farm itself.

## Multiple temporalities on the farm

The everyday practices on the farm may appear to a visitor as a continuous, year-round routine. Cows are milked and fed twice daily, and this cycle repeats without interruption, regardless of seasons, holidays, or market fluctuations. However, this apparent regularity is not self-sustaining; rather, it is made possible through the coordination of multiple intersecting rhythms. In the following subsections, I explain how this year-round daily repetition is enabled, adjusted, and sustained through the alignment of different temporalities: the cows’ biological rhythms, human labor routines, agricultural cycles, and market fluctuations. By unpacking these layered rhythms, I illustrate that what seems like a seamless cycle is, in fact, a precarious balance, requiring continuous interventions and adjustments to maintain.

### 24/7 lacto-cyclical temporality

The temporality of industrialized dairy production is defined by its 24/7 operation without interruption. This unceasing rhythm is sustained by the coordination of labor, technology, and the biological cycles of the cows. In this section, I present a typical workday on Ailin's farm to illustrate how dairy farming is structured around a continuous rhythm, operating year-round without interruption. The 24/7 lacto-cyclical temporality refers to the relentless, around-the-clock rhythm of dairy production, where cows, workers, and machines are synchronized to maintain continuous milk output.

**Figure 1.**
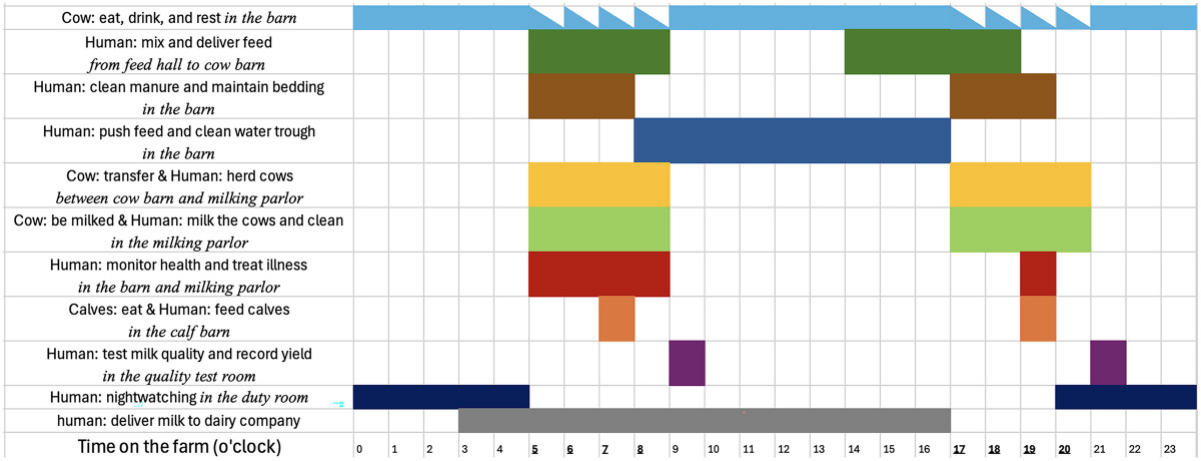
Around-the clock work routine on Ailin's farm. *The sawtooth pattern in the first row indicates the time when part of the herd is in the milking parlor, while the solid section shows when all cows are resting in the barn*.

As Figure 1 shows, Ailin's dairy farm operates on a tightly coordinated 24-h schedule, structured around two fixed daily milking shifts. The morning shift begins around 5 a.m. with a team of eight from the village: Aunt Hua, Aunt Ye, and Aunt Lu—three milkers in their 50s; Kang, the veterinarian; Laoshan, the accountant; Xiaoma, the feed mixer, Aunt Ning, who cleans the stalls, and Dahu, Ailin's husband, the manager. Kang and Laoshan herd cows in groups from the barn to the milking parlor, where the milkers await them and milk them in batches, starting with the high-yield cows, followed by the low-yield cows. Sick or newly calved cows are milked last, and their milk is set aside for feeding calves. During milking, Xiaoma prepares feed following Dahu's instructions and delivers customized feed to each group of cows, while Kang scrapes manure and Aunt Ning refreshes the bedding. Around 7 a.m., Aunt Hua takes two buckets of milk from the milking parlor to feed the calves, and then returns to the milking parlor to help clean the parlor with Aunt Ye and Aunt Lu. During milking, Laoshan records each cow's milk yield and enters the data into the system once milking ends. Dahu monitors every cow's data, including milk output, body condition, and reproductive status. He uses these metrics to make real-time adjustments to feeding and grouping decisions. The morning shift usually wraps up around 8:30 to 9:00 a.m.

By 8 a.m., Aunt Minghui arrives for her shift. She pushes feed closer to the cows, cleans the water troughs, and provides the cows with fresh water. After a midday break, Xiaoma resumes feed preparation around 2 p.m. in anticipation of the evening milking shift, which mirrors the morning's routine and ends around 9 p.m. From 9 p.m. to 5 a.m., Wang, the night-shift worker in his 60s, monitors the barn via surveillance, responding to escaped cows or overnight calvings. Around 4 a.m., Chengsi, the driver, arrives to transport the previous day's milk to Dingyao's factory, a 2-to-3-h drive. With many farms lining up to deliver milk, unloading takes time, so he usually returns by late afternoon. As 5 a.m. approaches, the daily cycle begins again.

The daily schedule described above highlights the extent to which modern dairy farming adheres to a strict, linear 24/7 milking schedule, structured entirely by shifts and routines. As Adam (1998: 140) points out, such practices exemplify the tendency to “decontextualize actions, processes, and phenomena” from their original ecological or biological contexts. Calves are separated from their mothers right after birth, and feeding is scheduled twice daily instead of following the calves’ natural tendency to suckle much more frequently. Within days, they stop responding to the outdoor mooing of adult cows, showing little sign of attachment. Adult cows’ feeding, milking, and reproduction are also meticulously calibrated by industrial timings, with workers subordinated to the logic of production. Workers described their routines as simply “work,” accepting early starts and work shifts as normal conditions of wage labor on the farm, reflecting what Thompson (1967) calls the internalization of industrial time-discipline. Ailin notes that if a worker takes leave, another villager can easily step in. These conditions reduce workers—cows and humans alike—to functional extensions of the production apparatus, their labor abstracted into mechanized routines instead of grounded in responsive embodiment, sustained through replacement when necessary. This logic becomes even more explicit in the regulation of cows’ reproduction, where productivity is embedded into their biology.

### Calibrated reproduction temporality

Sustaining steady herd reproduction is regarded as crucial for a farm's success in maintaining its lacto-cyclical rhythm. According to Dr Chen Kaixing, an expert in cattle farm management, a healthy herd should have around 10% of cows calving each month to maintain milk production levels. If the pregnancy rate falls below 20%, milk production will inevitably decrease (Nainiu Weikan, 2020). To maintain productivity, dairy cows must enter a continuous calving–insemination–pregnancy cycle, with success requiring careful attention at every stage of their fertility. Although cows naturally enter estrus throughout the year every 21 days, hormone injections are a common practice on industrial farms to synchronize cycles, enabling artificial insemination and predictable reproduction schedules. On the farm, reproduction is not merely biological but shaped by biotechnological interventions and economic decisions. A cow's value is tied to her ability to remain in this reproductive circuit, even as this exposes her to multiple painful health problems such as mastitis and mental distress in her shortened, precarious life (Karhu, 2024). Those unable to keep up are deemed expendable and sold off, underscoring how bovine life is continually measured against its economic utility.

In spring 2024, faced with falling milk and cow prices as well as rising feed costs, Ailin halted reproductive interventions. Kang noted that artificial insemination, once routine, was reserved only occasionally for young cows with strong reproductive potential. Besides, Ailin began restructuring her herd to reduce costs and adapt to shifting market conditions. In April and May 2024, she sold almost one-third of the herd, reducing the farm's size to around 360 cows. Most of the sold cows were calves and non-lactating cows, as they consumed resources without generating immediate returns. For Ailin, managing the cows is akin to allocating assets. One night in June, she walked me through her calculations: maintaining 200 lactating cows at moderate yield is more cost-effective than keeping a larger herd with non-producing members. Given the slim margins from milk alone, Ailin sees greater economic promise in retaining cows for sale when prices recover. As noted earlier, for managing feeding costs, Dahu tracks milk output daily, categorizing cows into high-yield and low-yield groups, with a tailored feed formula designed by Dingyao Dairy, with the high-yield feed being more costly. Dahu continuously assesses whether each cow's yield justifies her feeding cost, while keeping the heifers and calves as replacements for aging, lower-producing ones. This system, designed for efficiency, has grown increasingly fragile amid the current market downturn, with many small farms in rural Shijiazhuang already forced to close. To sustain her farm, Ailin reduced herd size and sold calves, while also continuing to plan future reproductive strategies in anticipation of improved market conditions.

The 24/7 lacto-cyclical rhythm of dairy farming relies on a tightly managed reproductive cycle, where cows must continuously calve to sustain milk production. This rhythm does not naturally occur but is actively coordinated through technological interventions, such as hormone injections and artificial insemination, to synchronize estrus cycles and ensure predictable calving intervals. Surveillance over cows’ bodies—monitoring milk yield, reproductive status, and overall condition—is a central and continuous practice, turning reproduction into a site of intensive calculation and control. However, Ailin's decision to halt these interventions amid falling milk prices reveals the fragility of this industrial temporality. Without a stable market or policy protection, farms like hers cannot rely on volume-based growth. Drawing on years of experience navigating cyclical fluctuations, Ailin aligns the cows’ biological rhythms with market expectations. Yet this strategy demands continuous adjustment and offers no guarantees. As many neighboring small farms shut down, Ailin's farm, like the cows she manages, can only survive by generating enough returns to sustain itself.

These adjustments are not without cost. For cows, they constantly endure physical and emotional strain for their whole life; for the farm, it requires constant vigilance to avoid falling out of the supply chain. The temporal coordination that sustains production is not stable or seamless, but contingent and extractive, assembling productivity through the manipulation of vulnerable bodies and the conditional survival of marginal farms, which requires meticulous calculation. In what follows, I turn to the situated practices, revealing how everyday adjustments in responsive interventions keep the industrial rhythm temporarily intact.

### Process temporality

Alongside the calibrated rhythms of reproduction, another form of temporality quietly structures the daily practices on Ailin's farm: a temporality of process, sustained through acts of care and repair. Reflecting on care work, Davies (1994: 280) contrasts task-oriented time with process time: the former abstracts discrete actions from their context, while the latter unfolds within and through social relations, moving along overlapping rhythms. Caring labor cannot be reduced to a single task or measured in discrete units of time; rather, it involves varied rhythms—repetitive and cyclical, linear and progressive, productive and sustaining, interrupted and forward-moving (Doucet, 2022; Maher, 2009). These rhythms intersect and intertwine, shaping practices that are scattered, interrelated, and extended across time and space. On the dairy farm, such forms of care become particularly visible in moments of disruption, when machines fail or cows fall ill. While certain problems are resolved through singular acts of technical repair, many require what Mol et al. (2010: 15) call “attuned attentiveness and adaptive tinkering”—a series of continuous, context-sensitive adjustments and material practices that respond to the situation as it unfolds.

In the milking parlor, 24 cows are milked at a time, arranged in two rows on either side of a narrow milking pit, where the milkers stand about 1 m below to operate the milking machines. Ideally, a screen above each milking unit displays real-time milk yield data that is automatically transmitted to a computer for record-keeping. However, due to a persistent malfunction, the data must be manually recorded. In each milking session, Laoshan first notes each cow's ear tag number after they have settled in the milking stall, then helps herd the next batch with Kang. He later returns to copy each cow's yield from the screen, except for six units that display no data. To fill the gaps, Laoshan estimates output based on past records, explaining, “Each cow doesn’t have a fixed milking position. If it's not recorded this time, there must be data from before. Just make an educated guess.” Once all cows are milked, Laoshan inputs all data into an old computer in the Quality Testing Room. One day, I took over the recording task, which required constant movement up and down the slippery milking pit to copy both ear tags and machine readouts. Aunt Hua commented, “We finished at least half an hour earlier. Usually, it takes much longer since Laoshan needs to walk the cows while also recording the data.” On top of that, Laoshan must enter all data into the system after the morning and afternoon sessions every day. Such labor requires not only bodily effort but also judgment and familiarity with the herd.

Laoshan's effort to compensate for the malfunctioning machine is tedious yet foreseeable. Caring for the herd's shifting biological states, however, demands constant vigilance and flexible responses. Each morning, Kang, the veterinarian, checks on the cows, but the milkers also monitor their health during milking. When a cow enters the milking station, her teats are disinfected, wiped clean, and then manually stripped three times. Milkers assess the texture and appearance of the milk. If something seems wrong, they test it with a reagent to check for signs of inflammation. Thickened milk indicates possible mastitis, in which case the cow is marked and transferred to the sick pen for treatment. Calves, more vulnerable to illness, especially diarrhea, require close daily observation. Aunt Hua watches them while feeding them and has developed ways to detect sickness. “Check the rear for wetness and watery manure,” she explained. “Sick calves usually lie down a lot. If one starts bouncing around, it's probably recovered.” Treatment practices are often carried out collectively rather than by the veterinarian alone. While Kang administers intravenous fluids and injections, Aunt Hua handles oral medications. With almost 20 years of experience raising cows at home, she adjusts treatments according to the calf's condition, often mixing gentamicin into their milk. Most calves make it through, but occasionally one dies.

Seasonal change brings additional layers to the process temporality. In May, as temperatures rise in North China, the sprinkling system is activated to keep the cows cool. When several hose nozzles had aged and needed fixing, the workers spontaneously cooperated: Kang and Xiaoma climbed up to replace the parts, while the milkers handed them tools. Such repair work is not assigned to anyone but emerges through shared attentiveness and mutual response.

On the farm, production continues not because everything functions smoothly, but because workers make it work through estimation, intuitive judgment, embodied skill, and trial-and-error adjustments. These moments of collaborative and consistent attentiveness reveal a temporal mode that is neither simply task-oriented nor linear but emergent and responsive. Rather than fitting neatly into standardized schedules, they reflect the inconsistencies of industrial dairy production and its reliance on constant care and repair. These practices are “nested” (Tronto, 2013: 21); they stretch across different actors, spaces, and moments, unfolding as an ongoing process to sustain the 24/7 rhythm of production. Some of these processual moments, such as fixing the sprinkling system, are prompted by seasonal shifts, that signal a deeper, cyclical temporality at work on the farm.

### Agricultural temporality guided by 节气 jieqi (the 24 solar terms)

In addition to the dairy farm's work schedule, the workers, mostly local farmers, also orient themselves around 节气 *jieqi*, the 24 solar terms of the Chinese agricultural calendar. The 节气 *jieqi* system divides the year into 24 equal segments, each marking a specific solar term such as 立春 *lichun* (Start of Spring), 雨水 *yushui* (Rainwater), and 谷雨 *guyu* (Grain Rain). These temporal markers signal phenological changes in the environment, providing practical guidance for agricultural activities. During my fieldwork, I notice that while 节气 *jieqi* has largely faded from daily awareness in urban and industrial contexts, it continues to structure farmers’ practices, shaping how they interpret weather and time their agricultural activities. For example, Aunt Minghui, the feed pusher, planted some cucumber vines next to the cowshed around 谷雨 *guyu* (late April to early May), explaining, “There's an old saying: ‘谷雨前后，种瓜种豆 *guyu qianhou, zhong gua zhong dou*’ (‘Around Grain Rain, plant melons and beans’).” Her casual reference demonstrated how 节气 *jieqi* persists to provide a practical guide for agricultural timing, persisting alongside the farm's industrial schedule. When I visited the farm again in late June, tiny cucumbers had already started to grow on the vines.

Although 节气 *jieqi* segments the year into 24 seasonal nodes and may seem unrelated to the daily rhythms of the dairy farm, it remains significant in subtle but persistent ways. During my fieldwork from early spring to mid-summer, I watched the surrounding landscape of the farm transform from bare soil to waist-high crops, a reminder that the farm's industrial routines remain deeply entangled with seasonal rhythms. This connection is particularly evident in silage, a significant portion of cows’ diet. With the help of microbial agents, corn stalks are harvested in early fall and stored as silage to feed the herd year-round. While cows on the farm do not graze on fresh grass—a practice more visibly dependent on seasonal and environmental rhythms—the corn still grows in synchrony with surrounding crops and is subject to the seasonal rhythms marked by 节气 *jieqi*. In spring 2024, a drought delayed corn planting and postponed the silage harvest from mid-September to early October. Over 3 days, Ailin harvested 1600 tons of stalks. According to Ailin, the harvest timing ultimately depends on the corn. In these moments, agricultural rhythms take the lead, and people follow. Silage thus embodies a layered temporality: it reflects the enduring influence of seasonal cycles while also exemplifying an industrial logic of control, wherein seasonal abundance is captured and redirected into a year-round, standardized feeding regime. Fermentation, as a multispecies temporal process (Siimes, 2025), involves microbial, vegetal, and human agencies. This microbial storage of time in silage further complicates the narrative of human mastery over nature, revealing how even attempts of control remain entangled with the very ecological processes it seeks to contain.

During my fieldwork, 节气 *jieqi* functions like the faint pulse of a timpani in an orchestra—subtle, rarely drawing direct attention, yet providing a steady undercurrent that shapes production. 节气 *Jieqi* has a cyclical ebb and flow such as spring planting, summer growth, fall harvest, and winter storage, that is aligned with natural seasonal processes. Meanwhile, work on the cow farm continues year-round, under a regimented industrial daily schedule shaped by technology, biomedicine, and market demands. Although these two systems operate at different paces, 节气 *jieqi* surfaces in moments such as the silage harvest or cucumber vines sprouting near the barn. While dairy farming follows clock time, one of its very cornerstones, silage production, remains tied to the seasonal rhythms of corn and the multispecies temporalities of fermentation. It exposes how industrial dairy is built upon and sustained by agricultural and ecological temporalities it seeks to standardize. In this, rather than serving as a passive backdrop, 节气 *jieqi* acts as a co-participant in sustaining dairy production (Plumwood, 2001).

## Concluding remarks

This paper has examined how industrial dairy production on Ailin's small-scale farm in China is temporally organized through the coordination of multiple, overlapping rhythms. Far from being seamless or self-sustaining, the 24/7 lacto-cyclical rhythm of milk production only emerges from constant alignment of heterogeneous temporalities across a more-than-human domain. These different rhythms are not simply biological or ecological patterns; they are made, maintained, and compensated for through labor, technological manipulation, and organizational strategies. What appears as a seamless routine is in fact a fragile assemblage.

To sustain this assemblage, both human and bovine bodies must be reoriented. The farm's operation relies on unbroken, round-the-clock labor across the entire calendar year. Workers reorganize their entire daily lives around the schedule without perceiving it as problematic despite its intensity. When one takes leave, another villager can step in. Their bodies become apparatuses of the system, and cows follow a similar logic. Twice a day, they are fed a nutritionally engineered formula, designed not around their individual needs but to optimize milk yield. They rest in between feedings, only to be milked at fixed times, also twice daily. Calves raised without their mothers lose the capacity for attachment, while mothers are treated as units in a high-efficiency production line.

Building on the daily reorientation of the cow's body around fixed routines, reproduction becomes another key site of intervention. On the farm, reproduction cycles are continuously calibrated, revealing an underlying logic of constant calculation and disposability. To ensure uninterrupted dairy production, cows must remain in a relentless cycle of pregnancy–birth–milking, aided by hormonal injections and artificial insemination. Once a cow becomes too exhausted from repeated pregnancies and milk extraction that her yield drops below the cost of feed, she is sold off, typically at the age of four to six, far short of her natural lifespan, which is 15 to 20 years (Karhu, 2024). In times of market fluctuation, this calibration intensifies at the herd level: entire groups of non-lactating cows are culled, revealing a managerial mode that treats the cows as economically adjustable and ultimately disposable.

This extractive system endures not through perfect synchronization but through ongoing, situated practices of care and repair (Mol et al., 2010). Workers attend closely to the cows by sensing changes in behavior and reading bodily signs. Machines, too, are not passive instruments of efficiency, but are kept running through tactile expertise and spontaneous collaboration (Bear and Holloway, 2019). Such unscheduled, often unrecognized gestures are not merely supplementary; they are what hold the system together when protocols fall short. It is the workers who compensate for the fragility of industrial rhythms by sustaining the process temporality through attentiveness, responsiveness, and embodied knowledge.

Yet while calibration, care and repair allow the system to endure on a daily basis, such efforts falter in the face of broader market shifts. Careful calibration on the farm, such as balancing of feed costs, insemination timing, and herd size, is rapidly upended as the operation scrambles to remain afloat. The logic of disposability that governs individual cows and herds now applies to the farm itself. This vulnerability is not incidental but structurally produced. Backed by both policy and capital investment, dominant dairy companies have expanded their operations from processing into breeding and farm ownership. This deepening vertical integration enables those capital-owned mega-farms to better withstand market shocks, while smaller farms like Ailin's are left to absorb the risks with little policy protection, functioning as buffers in times of oversupply (Liu, 2025). As a result, social farms across Hebei face mounting losses and are exiting at an accelerated pace. Within this shifting landscape, Ailin's strategies—selling cows, adjusting feed, halting reproduction—do not reflect mastery over time, but a precarious negotiation within it. Her pursuit of future profits mirrors the broader industry logic that seeks to resolve systemic instability through further acceleration. Yet as Berlant's (2011) critique of modernity in *Cruel Optimism* reminds us, this attachment to a promised future may, paradoxically, undermine the very conditions needed to realize it.

Still, some temporalities resist full control within this exhausted system. Industrial dairy production relies on ecological processes that unfold at their own pace. For example, successful silage production depends not on scheduling, but on agricultural rhythms shaped by soil, climate, fermentation, and human labor. These rhythms are not simply natural cycles, but ecological assemblages that are worked on, co-constituted, and only partially synchronized. They emerge within the system, unfolding through social relations and multispecies interactions. Neither task-oriented nor fully programmable, these temporalities are shaped by responsiveness and improvisation. In this sense, agricultural and processual rhythms share a common logic: they sustain production not by enforcing control, but by making room for relational coordination. Their fluidity and extensibility interrupt the standardizing tempo of extraction, carving out moments of interspecies intimacy and structural friction. These are not residual times, but essential temporalities that decelerate the system from within, exposing both its limits and the conditions for its persistence.

By providing a grounded ethnographic account of a small-scale farm on the periphery of industry production, this article reveals the multiple, often conflicting temporalities that sustain industrial dairy. Some of these temporalities are explicit and metrified; others are hidden, affective, and relational. Together, they expose the tensions within industrial coordination—between calibration and care, between disposability and maintenance. Calibration follows a logic of standardization and control, rendering both animals and farms interchangeable and disposable (Kosek, 2019: 167). Yet even calibration cannot escape the biological variance, seasonality, and ecological dependencies it seeks to dominate. Industrial dairy reorganizes these unruly rhythms but never fully controls them. Its apparent rigidity masks a precarious system held together by ongoing coordination across more-than-human domains. Care, by contrast, unfolds through situated acts that respond to breakdowns and maintain continuity (Mol et al., 2010). Though often overlooked and undervalued, this responsive labor helps hold the system together. Industrial dairy survives not through perfect synchronization, but through constant repair and compensation by those rendered marginal to its functioning: cows, human staff, ecologies, and small farms themselves.

Thinking temporally about industrial dairy production helps to reveal the hidden labor of synchronization and the ethical stakes involved: whose paces are prioritized; whose rhythms are disrupted; and who or what bears the cost of maintaining the illusion of efficiency and industrial coherence. As Adam (1995: 24) reminds us, industrial time tends to flatten complex biological and ecological rhythms subordinate to the logic of efficiency, but “there is no single time, only a multitude of times which interpenetrate and permeate our daily lives.” Recognizing this multiplicity is not merely a critique of industrial time, but an invitation to reorient our attention toward the fragile labor that makes coordination possible. As Gan and Tsing (2018: 116) suggest, rather than mastering, “attending is a good place to begin when thinking about temporal coordination.” On Ailin's farm, attending is not about restoring a seamless order, but about navigating the very frictions and limits that industrial time cannot erase. It means attuning to the multiplicity of rhythms—human and bovine, ecological and mechanical, and to the hidden labor and uneven costs that sustain the illusion of industrial coherence. In doing so, it gestures toward a temporal ethic grounded not in speed and scale, but in responsiveness, care, and relational coordination. This ethic affirms the value and potential of processual and ecological rhythms that resist standardization. In an industry strained by oversupply and contraction, such an orientation refuses the developmental logic of ever-larger, ever-faster production. Instead, it opens space to imagine futures where those at the margins are not rendered expendable, but recognized as indispensable to sustaining life.
